# Stability of Risk Perception Across Pandemic and Non-pandemic Situations Among Young Adults: Evaluating the Impact of Individual Differences

**DOI:** 10.3389/fpsyg.2022.840284

**Published:** 2022-02-24

**Authors:** Melissa T. Buelow, Jennifer M. Kowalsky, Amy B. Brunell

**Affiliations:** ^1^Department of Psychology, The Ohio State University, Newark, OH, United States; ^2^Department of Psychology, The Ohio State University, Mansfield, OH, United States

**Keywords:** risk perception, decision making, risk taking, personality, behavioral activation and inhibition, COVID-19

## Abstract

Previous research suggests a higher perceived risk associated with a risky behavior predicts a lower likelihood of involvement in that behavior; however, this relationship can vary based on personality characteristics such as impulsivity and behavioral activation. During the COVID-19 pandemic, individuals began to re-evaluate the level of risk associated with everyday behaviors. But what about risks associated with “typical” risk-taking behaviors? In the present study, 248 undergraduate student participants completed measures of impulsivity, behavioral activation and inhibition, propensity to take risks, numeracy, and perceptions of and involvement in both risk-taking behavior and health promoting behavior (e.g., blood donation, registering as an organ donor, vaccination). Our study revealed that higher behavioral inhibition and greater propensity to take risks predicted greater likelihood of involvement in COVID-19-related risk behaviors, even after accounting for perceived risks and benefits of the behavior. Greater likelihood of involvement in social risk behaviors was predicted by greater numeracy and risk-taking propensity. Identifying as male, a greater propensity to take risks, and greater impulsivity predicted increased health/safety risk behaviors. Younger age, lower risk-taking propensity, and lower impulsivity were associated with a greater likelihood of donating blood. For the likelihood of registering to become an organ donor, increasing risk perception, both before and during the pandemic, was associated with greater likelihood of registering, but greater risk-taking propensity was associated with a decreased likelihood of organ donation registration. For flu vaccination, a greater propensity to take risks was associated with a greater likelihood of flu vaccination during the 2020–2021 flu season. Both cognitive and personality factors can predict involvement in risk-taking and health-promotion behaviors, warranting their continued examination.

## Introduction

Individuals often engage in risk-taking behaviors, behaviors with the potential for a negative outcome for one’s health or wellbeing. These behaviors include use or overuse of substances (alcohol, tobacco, illicit drugs), speeding, and involvement in risky sexual behaviors. The decision to engage in these activities can come from a focus on immediate, short-term outcomes (typically rewards) at the expense of consideration of long-term outcomes (typically losses/risks) (e.g., Steinberg, 2008). Involvement in risk-taking behaviors activates the nucleus accumbens and other portions of the mesocorticolimbic (reward) pathway (Galvan et al., 2007), increasing the likelihood the behavior is repeated. Significant correlations are frequently found between self-reported involvement in different risk-taking behaviors (e.g., risky driving and substance use; Arnett et al., 1997; Bina et al., 2006; Antonopoulos et al., 2011; Gilman et al., 2015; Sween et al., 2017), although some find differences across domains (e.g., Nicholson et al., 2005; Friedman et al., 2014).

Cognitive and personality factors predict the likelihood of involvement in risk-taking behaviors. One such factor is risk perception, or one’s assessment of how risky an uncertain/ambiguous situation could be (Bettman, 1973; Baird and Thomas, 1985). Greater perceived benefits (Galvan et al., 2007; Soane et al., 2010; Hanoch et al., 2018) and lower perceived risks (Galvan et al., 2007; Mills et al., 2008; Soane et al., 2010; Gilman et al., 2015; Hanoch et al., 2018) predict greater involvement in risk-taking behaviors. Lower levels of numeracy, or the ability to understand probabilities and use this information to make decisions (Peters and Levin, 2008; Lipkus and Peters, 2009), also predict greater risk-taking (Wright et al., 2009; Brand et al., 2014; López-Pérez et al., 2017; Hanoch et al., 2018). In terms of personality, impulsivity (e.g., Donohew et al., 1999; Carlson et al., 2010; Braddock et al., 2011; Gilman et al., 2015; Maher et al., 2015; Reniers et al., 2016), sensation seeking (Dissabandara et al., 2014; Khodarahimi, 2015; Zhang et al., 2016), behavioral inhibition (BIS) and activation (BAS) (Nelson et al., 2008; Gullo et al., 2010; Braddock et al., 2011; Dissabandara et al., 2014; Reniers et al., 2016; Kemp et al., 2019; Blankenstein et al., 2020), and risk-taking propensity (Lejuez et al., 2004; Szrek et al., 2012; Markiewicz and Weber, 2013) predict involvement in risk-taking behaviors. However, in many of these studies, just one predictor, or one category of predictors, is typically examined and it is less clear how these potential predictors collectively predict risk taking. A comprehensive assessment of both cognitive and personality predictors is warranted.

A related issue is that risk perception and risk taking has shifted during the COVID-19 pandemic. Behaviors that were previously taken for granted, such as shaking hands or going grocery shopping, were now perceived as riskier to one’s health, and cognitive, demographic, and environmental factors affected risk perception (e.g., Cannito et al., 2020; Dryhurst et al., 2020; Liu et al., 2020; Alqahtani et al., 2021; Attema et al., 2021; Birhanu et al., 2021). For some individuals, risk-taking behaviors increased during the pandemic, as reflected in greater rates of speeding and substance use (e.g., Czeisler et al., 2020; Doucette et al., 2021a,b). For others, risk aversion driven by pandemic-related concerns increased, leading to lowered involvement in risky and greater involvement in health-promoting behaviors (e.g., increased hand hygiene; Shilo and Mor, 2020; Starks et al., 2020; Khan et al., 2021; Magnan et al., 2021; Shachat et al., 2021). Perceived risk of COVID-19 was associated with greater involvement in health-promoting behaviors that supported COVID-19 prevention (e.g., hand hygiene, maintaining social distance; Wise et al., 2020; Qin et al., 2021; Rui et al., 2021; Sinclair et al., 2021). Non-pandemic related health-promoting behaviors, such as blood donation and vaccination, were encouraged throughout the COVID-19 pandemic because, despite an element of risk, the benefits of these health-promoting behaviors outweighed potential costs.

Relatedly, the need for transfusions to save lives or improve quality of life (e.g., for people with sickle cell disease) continued throughout the COVID-19 pandemic, and alarm due to an unusually low blood supply was raised (American Red Cross, 2021). Motivators of blood donation behavior include perceived need, self-efficacy, and incentives, while deterrents include fear, low self-efficacy, and inconvenience (Bednall and Bove, 2011). Similarly, routine vaccinations were encouraged throughout the pandemic, with seasonal influenza vaccination strongly encouraged due to public health concern of co-circulation of influenza A (H3N2) and SARS-CoV-2 delta and omicron variants (Centres fot Diesease Control Prevention, 2021). Although blood donation and vaccination are safe overall, a risk of adverse events is present. Both are needle-related procedures and thus share risks, such as bruising at the procedure site and pre-faint symptoms (Kamel et al., 2010; McMurtry, 2020). These health-promoting behaviors share some of the same predictors as those of risk-taking behaviors. Greater perceived risks lower involvement in blood/organ donation behaviors (Allen and Butler, 1993; Norvilitis and Riley, 2001; Cohen, 2010; Mostafa, 2010; Chen, 2017), but *increase* the intent to vaccinate (e.g., Ferguson and Gallagher, 2007). Perceived procedural risk interacts with health messaging to produce a beneficial impact on intention to vaccinate (Ferguson and Gallagher, 2007). More specifically, among people with greater perceived risks of vaccination, exposure to health promotion messaging highlighting the vaccine effectiveness and the benefits missed out on if the vaccine is not obtained was associated with greater intention to vaccinate compared to those with lower perceived procedural risk.

The present study examines cognitive and personality predictors of involvement in both risk-taking and health-promoting behaviors within the context of the COVID-19 pandemic. In addition, we extend the current knowledge of predictors of vaccination and blood donation behaviors by assessing whether perceived risks associated with the behavior, in addition to personality and cognitive variables, predict involvement in the behavior. Specifically, we test the following hypotheses: (1) greater risk perception and numeracy, but lower impulsivity and behavioral activation, will predict lowered involvement in risk-taking behaviors; (2) greater risk perception related to COVID-19 will be associated with greater risk perception for other behaviors and predict lowered involvement in all risk-taking domains assessed; (3) self-reported perceived risk associated with getting a flu vaccine, donating blood, or registering as an organ donor will be higher when rated during COVID-19 than when retrospectively rated pre-COVID-19, and predict involvement in the behaviors; and (4) greater risk-taking propensity will predict greater vaccination and donation behaviors.

## Materials and Methods

### Participants

We completed several waves of data collection during the COVID-19 pandemic, all among undergraduate students who received course credit for participation in research studies. Participants were invited to complete the surveys in October 2020 and January 2021, corresponding to the beginning of the Fall and Spring semesters. At those times, the COVID-19 vaccine was not readily available, mask and distancing mandates were in place on campus, and the average University positivity rate was 0.81–2.14%. We allowed as many students as were interested to complete the study, leading to an initial sample of 272 participants. Participants were excluded from analyses due to missing data on multiple measures (*n* = 16) or reporting being interrupted a lot (*n* = 2), distracted a lot (*n* = 3), influenced by others (*n* = 2), or a combination of those factors (*n* = 1). Because risk perception varies by age, four participants over age 35 were excluded per American Psychological Association guidelines (VandenBos, 2007). Our final sample included 244 participants [ages 18–35 (*M* = 19.14, *SD* = 2.12), 98 males, 64.2% White, 23.0% Black or African American]. We conducted power analyses (G*Power; Faul et al., 2007) to determine the maximum effect size we were adequately powered to detect. Using a two-tailed test, this sample size could detect an effect size of *f^2^* = 0.12 (small-moderate) with a power of 0.95 and α = 0.05.

### Measures and Procedure

The study protocol was determined exempt from IRB review due to the anonymous survey design. All ethical guidelines were followed. Participants completed all measures online *via* a Qualtrics survey. After informed consent, participants completed a series of questionnaires in a randomized order that included the measures detailed below.

#### Personality Variables

The Barratt Impulsiveness Scale (BIS-11; Patton et al., 1995) assesses impulsivity as a personality construct via a series of 30 questions. Participants respond on a 1 (*Rarely/never*) to 4 (*Almost always/always*) scale. Average scores were calculated for the overall impulsiveness score (α = 0.809), with higher scores indicating greater levels of impulsiveness.

The BIS/BAS (Carver and White, 1994) assesses two competing systems: behavioral inhibition (BIS) and behavioral activation (BAS). Individuals high in BIS experience risk-avoidant behaviors in response to threats whereas individuals high in BAS instead engage in risk-seeking behaviors in response to reward signals. Participants responded to the 24 items on a 1 (*Very true for me*) to 4 (*Very false for me*) scale. Average scores were calculated for overall BIS (α = 0.799) and BAS (α = 0.810) scores, with higher scores indicating greater risk-avoidance (BIS) or risk-seeking (BAS).

#### Risk Perception Variables

The General Risk Propensity Scale (GRiPS; Zhang et al., 2019) assesses an individuals’ likelihood of taking risks across different situations. Participants responded to eight items on a 1 (*Strongly disagree*) to 6 (*Strongly agree*) scale. Two versions were administered: the standard GRiPS, which uses the term “In general,” at the beginning of each item (α = 0.913) and a modified GRiPS that instead used “During COVID-19” at the beginning of each item (α = 0.949). For each, an average score was calculated with higher values indicating greater propensity to take risks.

Participants also responded to a single-item assessment of general risk-taking, “*How willing are you to take risks, in general?*” on a 0 (*not at all willing*) to 10 (*very willing*) scale (Dohmen et al., 2011).

The expanded Lipkus numeracy scale (Lipkus et al., 2001) was administered. The measure assesses understanding of numbers, percentages, and relative risks, with three items assessing general numeracy and eight assessing risk numeracy. Each response was scored as correct or incorrect, then summed total scores were calculated for both general and risk numeracy.

#### Health Promotion and Risk-Related Variables

The Domain-Specific Risk Taking Scale (DOSPERT; Blais and Weber, 2006) was administered. Due to study time constraints, only the Social and Health/Safety subscales were utilized. A third subscale was developed to assess risk-taking during COVID-19 (see Kowalsky, in preparation for development information). For each subscale, participants responded to a series of risky situations in terms of (1) likelihood of involvement (1 = *Extremely unlikely*, 7 = *Extremely likely*), (2) perceived risks (1 = *Not at all risky*, 7 = *Extremely risky*), and (3) perceived benefits (1 = *No benefits at all*, 7 = *Great benefits*). Higher average scores indicated greater likelihood of involvement, greater perceived risks, and greater perceived benefits. Internal consistency was moderate to strong across subscales for likelihood of involvement (α = 0.635-0.890), perceived risks (α = 0.7052-0.952), and perceived benefits (α = 0.631-0.894).

Participants responded to a series of questions regarding their history of vaccination and donation behaviors. Specifically, participants were asked to indicate (0 = *no*, 1 = *yes*) whether they were vaccinated against the flu for the 2020–2021 season, ever donated blood, or ever registered to be an organ donor. Participants also indicated their perceived level of risk associated with each behavior. They used a 1 (*Not at all risky*) to 7 (*Extremely risky*) scale to indicate how much risk was associated with donating blood, registering to be an organ donor, and getting vaccinated against the seasonal flu, prior to (i.e., using retrospective self-report) and during the COVID-19 pandemic.

Finally, participants completed a questionnaire assessing demographic and background information. They then were debriefed and course credit was assigned. A portion of the participants completed follow-up testing (Time 2) at the end of each semester. Given the small sample size, these analyses are presented in Supplemental Analysis 1.

### Data Analysis

The hypotheses and analyses were registered at the Open Science Framework (OSF)^1^ following data collection but prior to analysis. To maximize power, we collapsed across the three BIS-11 subscale scores (Attentional Impulsivity, Motor Impulsivity, Non-planning Impulsivity) and the three BAS subscale scores (Drive, Fun Seeking, Reward Responsiveness) to create combined BIS-11 and BAS scores. This did not change the pattern of results, and the analyses by BIS-11 and BAS subscales can be found in Supplemental Analysis 2.

First, we assessed potential differences based on the time of data collection. The only significant differences emerged on behavioral inhibition, *t*(242) = 2.26, *p* = 0.012 (lower in Spring), and behavioral activation, *t*(242) = −2.99, *p* = 0.003 (higher in Spring). Therefore, a categorical variable for data collection time (1 = *Fall*, 2 = *Spring*) was added to the regression analyses. We also included age and gender as control variables, as risk perception can vary by these factors (e.g., Bonem et al., 2015; Brown et al., 2021), including during the COVID-19 pandemic (e.g., Alsharawy et al., 2021; Rosi et al., 2021).

Several blinded ex post analyses were conducted to assess the study hypotheses. First, Pearson correlations were examined to assess relationships between perceived risks and likelihood of involvement across DOSPERT risk-taking domains. Next, we conducted linear and logistic regressions examining whether risk and benefit perception, impulsivity, behavioral activation/inhibition, and numeracy predicted likelihood of involvement in risk-taking (DOSPERT domains) and health-promoting (blood donation, organ donation, vaccination) behaviors. Paired-samples *t*-tests were used to examine whether donating blood, registering to be an organ donor, or getting the flu vaccine were rated as riskier during COVID-19 than prior to COVID-19.

## Results

### Correlations

Means and standard deviations for all variables are included in Table 1. A correlation matrix (Table 2) indicated significant correlations between perceived risks, perceived benefits, and likelihood of involvement in different risk-taking behaviors. Specifically, positive correlations were found between perceived risks associated with social, health/safety, and COVID-19 behaviors (*r*s> 0.169, *p*s < 0.01). Positive correlations were also found between perceived benefits on the three DOSPERT subscales (*r*s > 0.170, *p*s < 0.01). Participants who reported a high likelihood of involvement in one DOSPERT risk-taking domain tended to also report a high likelihood of involvement in another DOSPERT domain (*r*s > 0.237, *p*s < 0.001). Thus, participants tended to interpret the risks and benefits of different types of risk-taking behaviors in a similar manner. Significant correlations were also found in the expected directions between impulsivity, BIS, BAS, and risk propensity (*r*s > 0.127, *p*s < 0.05), but not numeracy.

**TABLE 1:** Study variable means and standard deviations.

Variable	*n*	*M*	*SD*
BIS-11	244	2.07	0.33
BIS	244	3.03	0.56
BAS	244	3.04	0.41
Numeracy-general	243	1.30	1.07
Numeracy-risk	243	6.01	1.82
GRiPS-original	244	2.54	0.95
GRiPS-COVID	244	1.85	0.99
Dohmen	244	4.90	2.47
DOS-likelihood-social	243	4.43	1.00
DOS-likelihood-health	243	2.65	1.19
DOS-likelihood-COVID	242	4.11	1.19
DOS-risk-social	243	3.60	1.00
DOS-risk-health	243	5.65	1.02
DOS-risk-COVID	243	4.15	1.33
DOS-benefit-social	242	3.98	0.91
DOS-benefit-health	242	1.93	0.96
DOS-benefit-COVID	242	2.64	1.04
Risk blood donation: pre	242	1.95	1.41
Risk blood donation: COVID	241	3.45	1.90
Risk organ donation: pre	242	1.88	1.58
Risk organ donation: COVID	241	2.66	1.90
Risk flu vaccine: pre	242	2.04	1.50
Risk flu vaccine: COVID	241	2.93	1.78
Health promoting behaviors:			
Flu vaccine in 2020-2021	144 yes (59.3%)		
Donated blood	Ever: 155 yes (63.8%)		
Registered as organ donor	Ever: 99 yes (40.7%)		

*BIS-11, Barratt Impulsiveness Scale; BIS, behavioral inhibition from the BIS/BAS; BAS, behavioral activation from the BIS/BAS; GRiPS, General Risk Propensity Scale; DOS, Domain-Specific Risk Taking Scale.*

**TABLE 2:** Correlation matrix.

	1	2	3	4	5	6	7	8	9	10	11
1. Testing Time	−	0.108	–0.106	0.072	0.078	0.125	0.025	–0.041	–0.083	0.062	0.079
2. Age		−	−0.132*	0.121	0.069	–0.004	−0.149*	−0.143*	–0.070	0.053	–0.019
3. Gender			−	–0.123	−0.201**	–0.090	0.046	0.253***	0.134*	–0.048	−0.219***
4. DOSL-s				−	0.292***	0.238***	−0.227***	0.068	−0.141*	0.540***	0.014
5. DOSL-h					−	0.505***	0.027	−0.393***	−0.344***	0.192**	0.488***
6. DOSL-c						−	0.081	–0.040	−0.664***	0.194**	0.234***
7. DOSR-s							−	0.302***	0.170**	–0.067	0.193**
8. DOSR-h								−	0.279***	0.026	−0.382***
9. DOSR-c									−	–0.078	−0.153*
10. DOSB-s										−	0.171**
11. DOSB-h											−
12. DOSB-c											
13. BIS-11											
14. BIS											
15. BAS											
16. Num-G											
17. Num-R											
18. GRiPS											
19. GRiPS-C											
20. Dohmen											

	**12**	**13**	**14**	**15**	**16**	**17**	**18**	**19**	**20**		

1. Testing Time	0.135*	0.105	–0.101	0.129*	–0.060	0.059	0.100	0.105	0.061		
2. Age	0.020	–0.102	−0.151*	0.057	–0.055	0.070	0.060	–0.021	–0.002		
3. Gender	−0.191**	–0.096	0.370***	–0.039	−0.244***	−0.239***	−0.198**	−0.140*	–0.114		
4. DOSL-s	0.111	0.068	–0.111	0.142*	0.208**	0.259***	0.312***	0.170**	0.332***		
5. DOSL-h	0.345***	0.333***	–0.097	0.099	–0.038	–0.042	0.455***	0.427***	0.357***		
6. DOSL-c	0.578***	0.173**	0.089	0.114	0.057	0.046	0.343***	0.497***	0.322***		
7. DOSR-s	0.230***	0.161*	0.079	–0.038	–0.121	−0.137*	0.052	0.053	–0.010		
8. DOSR-h	−0.171**	−0.162*	0.175**	0.043	0.083	0.214***	−0.132*	−0.194**	–0.106		
9. DOSR-c	−0.476***	–0.083	–0.030	–0.113	–0.090	–0.063	−0.243***	−0.389***	−0.266***		
10. DOSB-s	0.285***	0.184**	0.026	0.103	0.006	0.108	0.236***	0.122	0.208**		
11. DOSB-h	0.574***	0.208**	−0.164*	–0.057	–0.067	−0.214***	0.265***	0.240***	0.240***		
12. DOSB-c	−	0.175**	–0.085	0.021	0.013	–0.075	0.331***	0.388***	0.252***		
13. BIS-11		−	0.105	0.089	–0.096	–0.071	0.278***	0.319***	0.144*		
14. BIS			−	0.003	–0.058	–0.013	−0.238***	–0.094	−0.156*		
15. BAS				−	0.044	0.035	0.273***	0.158*	0.242***		
16. Num-G					−	0.453***	0.087	0.043	0.069		
17. Num-R						−	0.133*	0.017	0.128*		
18. GRiPS							−	0.548***	0.746***		
19. GRiPS-C								−	0.482***		
20. Dohmen									−		

DOSL, DOSPERT likelihood of involvement in social (s), health/safety (h), and COVID-19 (c) behaviors; DOSR, DOSPERT perceived risks; DOSB, DOSPERT perceived benefits; BIS-11, Barratt Impulsiveness Scale; BIS, total behavioral inhibition from the BIS/BAS; BAS, total behavioral activation from the BIS/BAS; Num-G, General numeracy; Num-R, Risk-related numeracy; GRiPS, General Risk Propensity Scale, original and adapted for COVID (c). *p < 0.05. **p < 0.01. ***p < 0.001.

### Predicting Risk-Taking Behaviors

We next assessed whether perceived risk on the DOSPERT COVID-19 subscale predicted likelihood of involvement in health/safety, social, and COVID-related risk behaviors. Lower perceived risks predicted a greater likelihood of involvement in COVID-19-related, *F*(1, 240) = 189.37, *p* < 0.001, *R*^2^ = 0.441, *B* = −0.594, β = −0.664, health/safety, *F*(1,241) = 32.24, *p* < 0.001, *R*^2^ = 0.118, *B* = −0.308, β = 0.344, and social, *F*(1, 241) = 4.86, *p* = 0.028, *R*^2^ = 0.020, *B* = −0.106, β = −0.141, risk behaviors.

Next, linear regression analyses were conducted to examine whether perceived risks and benefits predicted likelihood of risk-taking behavior (see Table 3). For each analysis, age, gender (1 = *Male*, 2 = *Female*), and time of testing were entered in Step 1 as controls. Next, perceived risks and benefits for the specific set of behaviors were entered in Step 2 (behavior-specific perceived risks and benefits). Scores on the GRiPS, Dohmen, and numeracy scales were included in Step 3 (more generalized risk taking). Of note, we conducted two sets of regressions: one with the original GRiPS and one with the GRiPS modified for COVID-19. Finally, scores on the BIS-11 and BIS/BAS were entered in Step 4 (personality characteristics associated with risk taking). Collinearity statistics were within the reasonable range (Tolerance > 0.361, VIF < 2.767). Females reported a lower likelihood of involvement in health/safety behaviors only. Collectively, lower risk perception and greater benefit perception for COVID-19-related, health/safety, and social risk behaviors predicted greater likelihood of involvement in the associated behavior. After accounting for perceived risks and benefits, higher behavioral inhibition and propensity to take risks (adapted for COVID-19) predicted greater likelihood of involvement in COVID-19 risk behaviors. Greater likelihood of involvement in social risk behaviors was predicted by greater numeracy and greater risk-taking propensity. Finally, increased health/safety risk behaviors were predicted by greater propensity to take risks (both original and adapted for COVID-19) and greater impulsivity.

**TABLE 3:** Results of linear regression analyses predicting likelihood of engaging in COVID-19-related, social, and health/safety risky behaviors.

Analysis	Variable	*F*	△*R*^2^	B	β	Variable	*F*	△*R*^2^	B	β
		
	Analyses with GRiPS-original					Analyses with GRiPS-COVID				
**COVID-19**										
Step 1		1.20	0.015				1.20	0.015		
	Testing time			0.202	0.085	Testing time			0.202	0.085
	Age			–0.015	–0.027	Age			–0.015	–0.027
	Gender			–0.198	–0.082	Gender			–0.198	–0.082
Step 2		54.24***	0.521				54.24***	0.521		
	Risk-COVID			–0.455	−0.510***	Risk-COVID			–0.455	−0.510***
	Benefit-COVID			0.388	0.340***	Benefit-COVID			0.388	0.340***
Step 3		31.76***	0.017				34.58***	0.038		
	GRiPS			0.127	0.102	GRiPS-COVID			0.235	0.196***
	Dohmen			0.017	0.036	Dohmen			0.015	0.032
	Numeracy-gen			–0.007	–0.006	Numeracy-gen			–0.005	–0.005
	Numeracy-risk			0.027	0.042	Numeracy-risk			0.035	0.053
Step 4		24.85***	0.014				26.89***	0.012		
	BIS-11			0.106	0.029	BIS-11			0.009	0.002
	BIS			0.257	0.120*	BIS			0.258	0.121*
	BAS			0.020	0.007	BAS			0.035	0.012
**Social**										
Step 1		2.90*	0.035				2.90*	0.035		
	Testing time			0.145	0.073	Testing time			0.145	0.073
	Age			0.046	0.099	Age			0.046	0.099
	Gender			–0.235	–0.116	Gender			–0.235	–0.116
Step 2		24.45***	0.307				24.45***	0.307		
	Risk-social			–0.175	−0.175***	Risk-social			–0.175	−0.175***
	Benefit-social			0.569	0.519***	Benefit-social			0.569	0.519***
Step 3		18.86***	0.081				18.74***	0.080		
	GRiPS			0.068	0.065	GRiPS-COVID			0.017	0.017
	Dohmen			0.063	0.158*	Dohmen			0.079***	0.196***
	Numeracy-gen			0.125	0.135*	Numeracy-gen			0.126*	0.137*
	Numeracy-risk			0.050	0.092	Numeracy-risk			0.051	0.093
Step 4		14.12***	0.003				14.07***	0.003		
	BIS-11			0.008	0.003	BIS-11			0.024	0.008
	BIS			–0.105	–0.059	BIS			–0.116	–0.065
	BAS			0.035	0.014	BAS			0.044	0.018
**Health/Safety**										
Step 1		3.88*	0.047				3.88*	0.047		
	Testing time			0.114	0.048	Testing time			0.114	0.048
	Age			0.021	0.038	Age			0.021	0.038
	Gender			–0.477	−0.197**	Gender			–0.477	−0.197*
Step 2		19.66***	0.248				19.66***	0.248		
	Risk-health			–0.259	−0.221***	Risk-health			–0.259	−0.221***
	Benefit-health			0.488	0.392***	Benefit-health			0.488	0.392***
Step 3		17.28***	0.107				17.02***	0.104		
	GRiPS			0.426	0.339***	GRiPS-COVID			0.298	0.248***
	Dohmen			–0.001	–0.002	Dohmen			0.061	0.128*
	Numeracy-gen			–0.056	–0.051	Numeracy-gen			–0.057	–0.051
	Numeracy-risk			0.033	0.050	Numeracy-risk			0.043	0.065
Step 4		14.18***	0.025				13.92***	0.024		
	BIS-11			0.525	0.145*	BIS-11			0.548	0.151**
	BIS			0.129	0.060	BIS			0.063	0.029
	BAS			0.064	0.22	BAS			0.125	0.043

GRiPS, General Risk Propensity Scale, original and adapted for COVID; BIS-11, Barratt Impulsiveness Scale; BIS, total behavioral inhibition from the BIS/BAS; BAS, total behavioral activation from the BIS/BAS. *p < 0.05. **p < 0.01. ***p < 0.001.

### Predicting Health-Promoting Behaviors

We next examined blood donation, organ donation, and vaccination behaviors. First, paired-samples *t*-tests assessed the extent to which participants perceived risks associated with these behaviors as different before vs. during COVID-19. Participants reported that donating blood, *t*(240) = −12.38, *p* < 0.001, Cohen’s *d* = 0.797; registering as an organ donor, *t*(240) = −7.15, *p* < 0.001, *d* = 0.460; and vaccinating against the seasonal flu, *t*(240) = −7.96, *p* < 0.001, *d* = 0.513; were all riskier during compared to before the COVID-19 pandemic. We used these perceived risk ratings, both pre- and during COVID-19, as predictors of self-reported involvement in these activities (Table 4). Self-reported history of blood donation, registering as an organ donor, or getting the seasonal flu vaccine were coded as 0 (*No*) and 1 (*Yes*). The logistic regressions followed the same format at the DOSPERT regressions, with control variables entered in Step 1, perceived risks of the behavior in Step 2, risk-taking propensity variables in Step 3, and personality variables in Step 4.

**TABLE 4:** Results of logistic regression analyses predicting health promoting behaviors.

Analysis	Variable	χ ^2^	Nagelkerke *R*^2^	B	Wald	OR	Variable	χ ^2^	Nagelkerke *R*^2^	B	Wald	OR
		
	Analyses with GRiPS-Original						Analyses with GRiPS-COVID					
**Flu vaccine**												
Step 1		1.752	0.010					1.752	0.010			
	Testing time			–0.203	1.370	0.816	Testing time			–0.203	1.370	0.816
	Age			–0.028	0.188	0.973	Age			–0.028	0.188	0.973
	Gender			–0.140	0.243	0.869	Gender			–0.140	0.243	0.869
Step 2		4.405	0.036					4.405	0.036			
	Risk-pre			0.149	1.787	1.160	Risk-pre			0.149	1.787	1.160
	Risk-COVID			0.076	0.744	1.079	Risk-COVID			0.076	0.744	1.079
Step 3		9.963*	0.093					21.695***	0.156			
	GRiPS			0.201	0.719	1.222	GRiPS-COVID			0.661	10.691**	1.937
	Dohmen			–0.008	0.008	0.992	Dohmen			–0.067	0.885	0.935
	Numeracy-gen			0.164	1.086	1.178	Numeracy-gen			0.152	0.875	1.164
	Numeracy-risk			0.186	4.277*	1.205	Numeracy-risk			0.209	5.079*	1.233
Step 4		2.749	0.108					0.973	0.161			
	BIS-11			0.781	2.489	2.184	BIS-11			0.480	0.888	1.616
	BIS			–0.078	0.067	0.925	BIS			–0.065	0.046	0.937
	BAS			0.171	0.206	1.187	BAS			0.113	0.085	1.119
**Blood donation**												
Step 1		17.840***	0.101					17.840***	0.101			
	Testing time			0.379	4.104*	1.460	Testing time			0.379	4.104*	1.460
	Age			–0.266	10.381**	0.767	Age			–0.266	10.381**	0.767
	Gender			0.240	0.665	1.271	Gender			0.240	0.665	1.271
Step 2		0.578	0.104					0.578	0.104			
	Risk-pre			0.083	0.526	1.086	Risk-pre			0.083	0.526	1.086
	Risk-COVID			–0.009	0.012	0.991	Risk-COVID			–0.009	0.012	0.991
Step 3		4.121	0.126					4.591	0.129			
	GRiPS			–0.085	0.131	0.919	GRiPS-COVID			0.133	0.594	1.142
	Dohmen			–0.094	1.085	0.911	Dohmen			–0.144	4.317*	0.866
	Numeracy-gen			–0.033	0.044	0.967	Numeracy-gen			–0.036	0.050	0.965
	Numeracy-risk			0.015	0.027	1.015	Numeracy-risk			0.019	0.043	1.019
Step 4		6.565	0.161					7.875*	0.170			
	BIS-11			–0.788	2.494	0.455	BIS-11			–1.022	4.127*	0.360
	BIS			–0.347	1.229	0.707	BIS			–0.316	1.052	0.729
	BAS			0.522	1.903	1.685	BAS			0.499	1.781	1.648
**Organ donation**												
Step 1		1.688	0.010					1.688	0.010			
	Testing time			0.104	0.380	1.110	Testing time			0.104	0.380	1.110
	Age			–0.072	1.087	0.931	Age			–0.072	1.087	0.931
	Gender			–0.162	0.347	0.850	Gender			–0.162	0.347	0.850
Step 2		57.654***	0.302					57.654***	0.302			
	Risk-pre			0.327	6.995**	1.387	Risk-pre			0.327	6.995**	1.387
	Risk-COVID			0.430	20.471***	1.538	Risk-COVID			0.430	20.471***	1.538
Step 3		3.611	0.318					4.838	0.323			
	GRiPS			0.017	0.005	1.018	GRiPS-COVID			0.198	1.230	1.219
	Dohmen			–0.124	1.641	0.883	Dohmen			–0.158	4.595*	0.854
	Numeracy-gen			0.004	0.001	1.004	Numeracy-gen			0.000	0.000	1.000
	Numeracy-risk			0.031	0.105	1.031	Numeracy-risk			0.038	0.156	1.038
Step 4		4.539	0.337					4.848	0.344			
	BIS-11			–0.122	0.052	0.885	BIS-11			–0.336	0.385	0.715
	BIS			–0.613	3.751	0.542	BIS			–0.594	3.606	0.552
	BAS			0.263	0.442	1.301	BAS			0.241	0.374	1.273

χ^2^ and Nagelkerke R^2^ values are for the current step, not the overall model. GRiPS, General Risk Propensity Scale, original and adapted for COVID; BIS-11, Barratt Impulsiveness Scale; BIS, total behaviora inhibition from the BIS/BAS; BAS, total behavioral activation from the BIS/BAS. *p < 0.05. **p < 0.01. ***p < 0.001.

For a history of blood donation, the overall regression model, using the GRiPS adapted for COVID, was significant, χ^2^(12) = 30.885, *p* = 0.002, and explained 17.0% (Nagelkerke *R*^2^) of the variance while correctly classifying 67.5% of cases. Completing the survey in January, younger age, and lower risk propensity and impulsivity were associated with a greater likelihood of donating blood. Except for the Dohmen and impulsivity, these relationships held when the original GRiPS was included in the model.

The overall logistic regression model, using the GRiPS adapted for COVID, was significant for organ donation registration behavior, χ^2^(12) = 65.446, *p* < 0.001, and explained 33.5% (Nagelkerke *R*^2^) of the variance while correctly classifying 73.8% of cases. Increasing risk perception, both pre- and during COVID, was associated with a greater likelihood of registering to be an organ donor. In addition, greater risk-taking propensity (Dohmen) was associated with a decreased likelihood of registering to be an organ donor. Except for the Dohmen, these relationships held when the original GRiPS was included in the model.

The overall model for flu vaccination behaviors, when the original GRiPS was in the model, was not significant, χ^2^(12) = 18.868, *p* = 0.092, Nagelkerke *R*^2^ = 0.108. When the GRiPS-COVID-19 adaptation was utilized, the overall model was significant, χ^2^(12) = 28.825, *p* = 0.004, Nagelkerke *R*^2^ = 0.161, and correctly classified 65.9% of participants. A greater propensity to take risks, as assessed by the GRiPS adapted for COVID-19, and greater numeracy were associated with a greater likelihood of flu vaccination during the 2020–2021 season.

## Discussion

This study examined cognitive and personality predictors of risk-taking and health-promoting behaviors during the COVID-19 pandemic. Other research demonstrated both increased risk-taking (e.g., Czeisler et al., 2020; Doucette et al., 2021a,b) and risk aversion (e.g., Shilo and Mor, 2020; Starks et al., 2020; Khan et al., 2021; Magnan et al., 2021; Shachat et al., 2021) during the pandemic, and we examined the extent to which previously identified predictors of risk predicted risk-taking during a pandemic.

First, we assessed whether greater risk perception and numeracy, but lower impulsivity and BAS, predicted lower involvement in risk-taking behaviors. Our results partially support this hypothesis. Perceived risks and benefits were the most consistent predictors of risk-taking. Across health/safety, social, and COVID-19-related behaviors, participants were more likely to take a risk if they endorsed low risk and high benefit perception, consistent with research prior to the pandemic (Galvan et al., 2007; Soane et al., 2010; Hanoch et al., 2018). After accounting for these factors, participants endorsing a high risk-taking propensity endorsed a greater likelihood of involvement in all risk-taking behaviors assessed. The influence of the other predictors depended on the type of behavior examined: (1) greater BIS predicted greater COVID-19-related risk-taking, (2) greater numeracy predicted greater social risk-taking, and (3) greater impulsivity predicted greater health/safety-related risk-taking. These results are consistent with some previous research indicating more impulsive individuals take more risks (Donohew et al., 1999; Carlson et al., 2010; Braddock et al., 2011; Gilman et al., 2015; Maher et al., 2015; Reniers et al., 2016), but counter those finding lower BIS (Braddock et al., 2011; Maher et al., 2015), lower numeracy (Brand et al., 2014; Hanoch et al., 2018), and greater BAS (Gullo et al., 2010; Dissabandara et al., 2014; Blankenstein et al., 2020) predict greater risk-taking behavior. Although further research is warranted to assess these relationships, it is possible that participants who experienced greater risk aversion (high BIS) but also a greater likelihood of COVID-19-related risk-taking behavior did not view the behaviors as particularly risky, or at least not risky enough to signal aversion to the behavior. The positive relationship between numeracy and social risk-taking runs counter to previous research and to expectation; however, examination of the specific DOSPERT items that make up this scale may play a role. While the health/safety and COVID-19-related DOSPERT items focus on behaviors that could be riskier during COVID-19 (e.g., unprotected sex, overuse of alcohol), the social subscale items do not rely on an in-person, physical interaction with someone (e.g., disagreeing with someone, speaking up in a meeting). It is possible that participants, including those who are knowledgeable about probabilities, recognized the low risk of COVID-19 in these situations. It is also possible that more numerate participants took greater risks due to how they weighed risks vs. benefits, in keeping with previous research indicating that the relative weighting of these factors changes the decision to take risks (e.g., Reyna and Farley, 2006; Maslowsky et al., 2011; Reyna et al., 2011). But in our findings, numeracy did not predict involvement in health/safety or COVID-19-related behaviors, and future research should investigate this anomaly.

We next examined relationships between COVID-19-related risk perception and involvement in different risk behaviors, and whether changing risk perception might affect both risk-taking and health-promoting behaviors. The results supported our hypothesis, as participants reporting greater COVID-19-related risk perception also rated social and health/safety behaviors as riskier. In terms of health-promoting behaviors, participants reported that donating blood, registering as an organ donor, and vaccinating against the seasonal flu were all riskier during vs. prior to the pandemic. Risk perception also affected the likelihood of registering as an organ donor but not of engaging in vaccination or blood donation behaviors. A greater risk-taking propensity predicted increased likelihood of vaccination but decreased likelihood of blood and organ donation. The only personality predictor to emerge was impulsivity, as less impulsivity was associated with greater likelihood of blood donation. Taken together, we find relatively little evidence that the personality variables previously shown to predict involvement in risk-taking behavior predict these health-promoting behaviors.

There are several limitations that could affect generalizability of the findings. Not all areas of risk-taking or health-promoting behavior were assessed. It is possible that personality and cognitive variables differentially predict involvement in other risk-taking behaviors. In addition, we did not conduct an exhaustive assessment of all cognitive or personality predictors; thus, future research should assess other facets that may affect risk perception. We utilized a convenience sample, and it is unclear if these same factors would predict behavior among older or more diverse individuals. As the relationship between factors is complex, future research should utilize structural equation modeling techniques in these larger and more diverse samples. Finally, we relied on self-report of both the predictors and of risk-taking behaviors. Although many previous studies have utilized the DOSPERT to assess risk, future studies should pair behavioral with self-report measures to assess the construct more fully.

Taken together, the present study provides evidence that cognitive and personality factors predict involvement in risk-taking and health-promoting behaviors, but the effect varies by the particular behavior studied. Future research examining risk-taking and health promotion should continue to assess different behaviors, as targets of interventions may depend on the particular behavior. Risk perception appears to differ based on the context of the behavior/situation, as can how cognitive and personality variables affect this relationship. From a health behavior perspective, individuals rated health behaviors with an interpersonal element, such as donating blood or receiving a vaccine, as more risky during COVID-19 vs. pre-pandemic. Although we relied on retrospective self-report of estimated risk, future research should examine longitudinally how risk perception changes as we emerge from the pandemic. During COVID-19, there has been an increased need for both blood donation and vaccination, as both behaviors support the individual and the community as a whole. Moving forward, there will likely be a need to address novel risk-related concerns within local medical settings (e.g., strategies in place to mitigate spread of a virus, how these strategies are communicated to reduce risk perception). Conveying important health-related information in a way that increases the likelihood of health-promoting and decreases the likelihood of risk-taking behaviors is vital to a healthy community.

## Data Availability Statement

The datasets presented in this study can be found in online repositories. The names of the repository/repositories and accession number(s) can be found below: https://osf.io/aetfc/?view_only=20b3376236244f40a9a810303938dab3.

## Ethics Statement

Ethical review and approval was not required for the study on human participants in accordance with the local legislation and institutional requirements. The patients/participants provided their informed consent to participate in this study.

## Author Contributions

MB, JK, and AB designed the study and contributed to the drafting and revision of the manuscript. MB collected and analyzed the data. All authors contributed to the article and approved the submitted version.

## Conflict of Interest

The authors declare that the research was conducted in the absence of any commercial or financial relationships that could be construed as a potential conflict of interest.

## Publisher’s Note

All claims expressed in this article are solely those of the authors and do not necessarily represent those of their affiliated organizations, or those of the publisher, the editors and the reviewers. Any product that may be evaluated in this article, or claim that may be made by its manufacturer, is not guaranteed or endorsed by the publisher.

## Footnotes

1
https://osf.io/aetfc/?view_only=20b3376236244f40a9a810303938dab3
