# Supplementary material for: Stability of Risk Perception Across Pandemic and Non-pandemic Situations Among Young Adults: Evaluating the Impact of Individual Differences
Source: Front Psychol. 2022 Feb 24;13:840284. doi: 10.3389/fpsyg.2022.840284 (PMC8907664; doi:10.3389/fpsyg.2022.840284)
Supplement: Supplementary file 1 [file Data_Sheet_1.docx]

Supplemental Analysis 1: Time 1 Variables Predicting Time 2 Behaviors

Undergraduate student participants were given the opportunity to complete a second administration of a subset of the study tasks at the end of the Fall 2020 (November) or Spring 2021 (March) semester. Of the 244 participants who completed all Time 1 tasks, 52 fully completed the Time 2 study (ages 18-35 [*M*=19.59, *SD*=2.94], 22 males, 66.7% White, 24.1% Black or African American). At Time 2, participants completed the following measures: DOSPERT COVID-19 subscale, Dohmen, risks of health-promoting behaviors, vaccination and donation behaviors, GRiPS-COVID-19.

We conducted a series of analyzes assessing the extent to which risk perception for COVID-19-related behaviors changed from Time 1 to Time 2, and whether predictors assessed at Time 1 could predict likelihood of involvement in these behaviors at Time 2. Paired-samples t-tests indicated no differences in perceived risks (*t*[51]=1.799, *p*=.078) or involvement (*t*[51]=0.093, *p*=.926) in COVID-19-risk behaviors, nor in overall risk-taking propensity with the Dohmen (*t*[51]=-1.467, *p*=.149) or GRiPS-COVID-19 adaptation (*t*[50]=-0.671, *p*=.505). Thus, perceived risks associated with COVID-19 did not increase nor decrease as the pandemic continued. A linear regression was conducted with Time 1 predictors of Time 2 likelihood of involvement in COVID-19 risk behaviors (Table 1). Greater perceived benefits of COVID-19 related behaviors, assessed at Time 1, predicted greater involvement in these risky behaviors at Time 2.

**Supplemental Table 1**. Results of Time 2 linear regression analysis.

| Analysis | Variable | F | $\Delta$*R^2^* | B | $\beta$ | Variable | F | $\Delta$*R^2^* | B | $\beta$ |
| --- | --- | --- | --- | --- | --- | --- | --- | --- | --- | --- |
|  | Analyses with GRiPS-Original | | | | | Analyses with GRiPS-COVID | | | | |
| *COVID-Time 2* |  |  |  |  |  |  |  |  |  |  |
| Step 1 |  | 2.943^*^ | .155 |  |  |  | 2.943^*^ | .155 |  |  |
|  | Testing Time |  |  | -0.020 | -0.009 | Testing Time |  |  | -0.020 | -0.009 |
|  | Age |  |  | 0.044 | 0.122 | Age |  |  | 0.044 | 0.122 |
|  | Gender |  |  | -0.793 | -0.362^*^ | Gender |  |  | -0.793 | -0.362^*^ |
| Step 1 |  | 9.231^***^ | .345 |  |  |  | 9.231^***^ | .345 |  |  |
|  | Risk-COVID |  |  | -0.214 | -0.256 | Risk-COVID |  |  | -0.214 | -0.256 |
|  | Benefit-COVID |  |  | 0.429 | 0.428^**^ | Benefit-COVID |  |  | 0.429 | 0.428^**^ |
| Step 2 |  | 6.997^***^ | .099 |  |  |  | 7.065^***^ | .101 |  |  |
|  | GRiPS |  |  | 0.030 | 0.028 | GRiPS-COVID |  |  | 0.065 | 0.069 |
|  | Dohmen |  |  | 0.107 | 0.230 | Dohmen |  |  | 0.100 | 0.216 |
|  | Numeracy-Gen |  |  | -0.091 | -0.088 | Numeracy-Gen |  |  | -0.098 | -0.096 |
|  | Numeracy-Risk |  |  | 0.116 | 0.183 | Numeracy-Risk |  |  | 0.121 | 0.192 |
| Step 3 |  | 5.557^***^ | .031 |  |  |  | 5.658^***^ | .033 |  |  |
|  | BIS-11 |  |  | 0.263 | 0.088 | BIS-11 |  |  | 0.216 | 0.072 |
|  | BIS |  |  | 0.121 | 0.058 | BIS |  |  | 0.147 | 0.071 |
|  | BAS |  |  | -0.446 | -0.164 | BAS |  |  | -0.475 | -0.175 |
|  |  |  |  |  |  |  |  |  |  |  |

Supplemental Analysis 2: Analyses with the BIS-11 and BIS/BAS Subscale Scores

The following tables and results are for analyses with the three BIS-11 subscale scores (Attention Impulsiveness, Motor Impulsiveness, Nonplanning Impulsiveness) and the three BAS subscale scores from the BIS/BAS (Drive, Fun Seeking, Reward Responsiveness). The overall pattern of results from the linear regressions followed that when the BIS-11 and BAS composite scores were utilized. Greater involvement in COVID-19-related risk behaviors was predicted by lower perceived risks, greater perceived benefits, and greater risk propensity (specific to COVID-19). Greater involvement in social risk behaviors was predicted by lower perceived risks, greater perceived benefits, greater risk propensity, and greater numeracy. Finally, greater involvement in health-safety risk behaviors was predicted by lower perceived risks, greater perceived benefits, greater risk propensity (in general and specific to COVID-19), and greater BIS-attentional impulsivity.

**Supplemental Table 2.** Means, standard deviations, and correlation matrix.

|  | M | SD | 1 | 2 | 3 | 4 | 5 | 6 | 7 | 8 | 9 | 10 | 11 | 12 | 13 |
| --- | --- | --- | --- | --- | --- | --- | --- | --- | --- | --- | --- | --- | --- | --- | --- |
| 1. Testing Time | -- | -- | -- | .147^*^ | -.120 | -.054 | .007 | .108 | -.144^*^ | .175^**^ | .188^**^ | .088 | -.077 | .075 | .106 |
| 2. Age | 19.14 | 2.12 |  | -- | -.132^*^ | -.076 | -.069 | -.095 | -.151^*^ | .116 | -.035 | .043 | -.055 | .070 | .060 |
| 3. Gender | -- | -- |  |  | -- | -.038 | -.075 | -.107 | .370^***^ | -.160^*^ | -.017 | .089 | -.244^***^ | -.239^***^ | -.198^**^ |
| 4. BIS-11-AI | 2.23 | 0.48 |  |  |  | -- | .379^***^ | .429^***^ | .192^**^ | -.009 | .186^**^ | .032 | -.013 | .009 | .150^*^ |
| 5. BIS-11-MI | 1.91 | 0.34 |  |  |  |  | -- | .503^***^ | -.047 | .193^**^ | .321^***^ | -.053 | -.035 | -.109 | .393^***^ |
| 6. BIS-11-NPI | 2.12 | 0.43 |  |  |  |  |  | -- | .099 | -.080 | .170^**^ | -.131^*^ | -.159^*^ | -.068 | .141^*^ |
| 7. BIS | 3.03 | 0.56 |  |  |  |  |  |  | -- | -.291^***^ | .055 | .253^***^ | -.058 | -.013 | -.238^***^ |
| 8. BAS-D | 2.68 | 0.58 |  |  |  |  |  |  |  | -- | .494^***^ | .345^***^ | .019 | -.011 | .304^***^ |
| 9. BAS-F | 2.93 | 0.50 |  |  |  |  |  |  |  |  | -- | .479^***^ | .033 | .007 | .331^***^ |
| 10. BAS-R | 3.43 | 0.47 |  |  |  |  |  |  |  |  |  | -- | .053 | .084 | .026 |
| 11. Num-G | 1.30 | 1.07 |  |  |  |  |  |  |  |  |  |  | -- | .453^***^ | .087 |
| 12. Num-R | 6.02 | 1.81 |  |  |  |  |  |  |  |  |  |  |  | -- | .133^*^ |
| 13. GRiPS | 2.53 | 0.95 |  |  |  |  |  |  |  |  |  |  |  |  | -- |
| 14. GRiPS-C | 1.84 | 0.99 |  |  |  |  |  |  |  |  |  |  |  |  |  |
| 15. Dohmen | 4.88 | 2.48 |  |  |  |  |  |  |  |  |  |  |  |  |  |
| 16. DOS-L-s | 4.44 | 1.03 |  |  |  |  |  |  |  |  |  |  |  |  |  |
| 17. DOS-L-h | 2.64 | 1.19 |  |  |  |  |  |  |  |  |  |  |  |  |  |
| 18. DOS-L-c | 4.08 | 1.20 |  |  |  |  |  |  |  |  |  |  |  |  |  |
| 19. DOS-R-s | 3.57 | 1.02 |  |  |  |  |  |  |  |  |  |  |  |  |  |
| 20. DOS-R-h | 5.64 | 1.02 |  |  |  |  |  |  |  |  |  |  |  |  |  |
| 21. DOS-R-c | 4.17 | 1.33 |  |  |  |  |  |  |  |  |  |  |  |  |  |
| 22. DOS-B-s | 3.98 | 0.91 |  |  |  |  |  |  |  |  |  |  |  |  |  |
| 23. DOS-B-h | 1.92 | 0.96 |  |  |  |  |  |  |  |  |  |  |  |  |  |
| 24. DOS-B-c | 2.63 | 1.03 |  |  |  |  |  |  |  |  |  |  |  |  |  |

**p* < .05

***p* < .01

****p* < .001

*Note*: BIS-11 = Barratt Impulsiveness Scale, Attentional (AI), Motor (MI), and Nonplanning (NPI) impulsiveness; BIS = BIS from the BIS/BAS; BAS = BAS drive (D), fun seeking (F), and reward responsiveness (R) from the BIS/BAS; Num-G = General numeracy; Num-R = Risk-related numeracy; DOS-L = DOSPERT likelihood of involvement in social (s), health/safety (h), and COVID-19 (c) behaviors; DOS-R = DOSPERT perceived risks; DOS-B = DOSPERT perceived benefits

|  | 14 | 15 | 16 | 17 | 18 | 19 | 20 | 21 | 22 | 23 | 24 |
| --- | --- | --- | --- | --- | --- | --- | --- | --- | --- | --- | --- |
| 1. Testing Time | .091 | .089 | .114 | .087 | .074 | -.037 | -.041 | -.043 | -.012 | .024 | .083 |
| 2. Age | -.021 | -.002 | .121 | .069 | -.004 | -.149^*^ | -.143^*^ | -.070 | .053 | -.019 | .020 |
| 3. Gender | -.140^*^ | -.114 | -.123 | -.201^**^ | -.090 | .046 | .253^***^ | .134^*^ | -.048 | -.219^***^ | -.191^**^ |
| 4. BIS-AI | .158^*^ | .068 | .087 | .256^***^ | .121 | .216^***^ | -.054 | -.086 | .149^*^ | .133^*^ | .131^*^ |
| 5. BIS-MI | .365^***^ | .263^***^ | .104 | .337^***^ | .206^**^ | .092 | -.182^**^ | -.084 | .159^*^ | .263^***^ | .235^***^ |
| 6. BIS-NPI | .240^***^ | .034 | -.013 | .213^***^ | .097 | .087 | -.147^*^ | -.036 | .133^*^ | .113 | .070 |
| 7. BIS | -.094 | -.156^*^ | -.111 | -.097 | .089 | .079 | .175^**^ | -.030 | .026 | -.164^*^ | -.085 |
| 8. BAS-D | .188^**^ | .190^**^ | .137^*^ | .169^**^ | .040 | -.027 | -.108 | -.076 | .103 | .125 | .074 |
| 9. BAS-F | .224^***^ | .307^***^ | .115 | .178^**^ | .195^**^ | -.003 | -.006 | -.115 | .075 | .023 | .088 |
| 10. BAS-R | -.024 | .092 | .085 | -.100 | .050 | -.056 | .211^***^ | -.082 | .065 | -.275^***^ | -.103 |
| 11. Num-Gen | .043 | .069 | .208^**^ | -.038 | .057 | -.121 | .083 | -.090 | .006 | -.067 | .013 |
| 12. Num-Risk | .017 | .128^*^ | .259^***^ | -.042 | .046 | -.137^*^ | .214^***^ | -.063 | .108 | -.214^***^ | -.075 |
| 13. GRiPS | .548^***^ | .746^***^ | .312^***^ | .455^***^ | .343^***^ | .052 | -.132^*^ | -.243^***^ | .236^***^ | .265^***^ | .331^***^ |
| 14. GRiPS-C | -- | .482^***^ | .170^**^ | .427^***^ | .497^***^ | .053 | -.194^**^ | -.389^***^ | .122 | .240^***^ | .388^***^ |
| 15. Dohmen |  | -- | .332^***^ | .357^***^ | .322^***^ | -.010 | -.106 | -.266^***^ | .208^**^ | .240^***^ | .252^***^ |
| 16. DOS-L-s |  |  | -- | .292^***^ | .238^***^ | -.227^***^ | .068 | -.141^*^ | .540^***^ | .014 | .111 |
| 17. DOS-L-h |  |  |  | -- | .505^***^ | .027 | -.393^***^ | -.344^***^ | .192^**^ | .488^***^ | .345^***^ |
| 18. DOS-L-c |  |  |  |  | -- | .081 | -.040 | -.664^***^ | .194^**^ | .234^***^ | .578^***^ |
| 19. DOS-R-s |  |  |  |  |  | -- | .302^***^ | .170^**^ | -.067 | .193^**^ | .230^***^ |
| 20. DOS-R-h |  |  |  |  |  |  | -- | .279^***^ | .026 | -.382^***^ | -.171^**^ |
| 21. DOS-R-c |  |  |  |  |  |  |  | -- | -.078 | -.153^*^ | -.476^***^ |
| 22. DOS-B-s |  |  |  |  |  |  |  |  | -- | .171^**^ | .285^***^ |
| 23. DOS-B-h |  |  |  |  |  |  |  |  |  | -- | .574^***^ |
| 24. DOS-B-c |  |  |  |  |  |  |  |  |  |  | -- |

**Supplemental Table 3**. Results of linear regression analyses.

| Analysis | Variable | F | $\Delta$*R^2^* | B | $\beta$ | Variable | F | $\Delta$*R^2^* | B | $\beta$ |
| --- | --- | --- | --- | --- | --- | --- | --- | --- | --- | --- |
|  | Analyses with GRiPS-Original | | | | | Analyses with GRiPS-COVID | | | | |
| *COVID* |  |  |  |  |  |  |  |  |  |  |
| Step 1 |  | 1.022 | .013 |  |  |  | 1.022 | .013 |  |  |
|  | Testing Time |  |  | 0.168 | 0.071 | Testing Time |  |  | 0.168 | 0.071 |
|  | Age |  |  | -0.014 | -0.025 | Age |  |  | -0.014 | -0.025 |
|  | Gender |  |  | -0.200 | -0.083 | Gender |  |  | -0.200 | -0.083 |
| Step 2 |  | 54.195^***^ | .523 |  |  |  | 54.195^***^ | .523 |  |  |
|  | Risk-COVID |  |  | -0.456 | -0.510^***^ | Risk-COVID |  |  | -0.456 | -0.510^***^ |
|  | Benefit-COVID |  |  | 0.389 | 0.340^***^ | Benefit-COVID |  |  | 0.389 | 0.340^***^ |
| Step 3 |  | 31.733^***^ | .017 |  |  |  | 34.551^***^ | .038 |  |  |
|  | GRiPS |  |  | 0.126 | 0.101 | GRiPS-COVID |  |  | 0.235 | 0.196^***^ |
|  | Dohmen |  |  | 0.017 | 0.036 | Dohmen |  |  | 0.015 | 0.032 |
|  | Numeracy-Gen |  |  | -0.007 | -0.006 | Numeracy-Gen |  |  | -0.006 | -0.005 |
|  | Numeracy-Risk |  |  | 0.028 | 0.043 | Numeracy-Risk |  |  | 0.035 | 0.054 |
| Step 4 |  | 18.836^***^ | .021 |  |  |  | 20.442^***^ | .020 |  |  |
|  | BIS-AI |  |  | -0.098 | -0.039 | BIS-AI |  |  | -0.082 | -0.033 |
|  | BIS-MI |  |  | 0.173 | 0.050 | BIS-MI |  |  | 0.126 | 0.037 |
|  | BIS-NPI |  |  | 0.004 | 0.002 | BIS-NPI |  |  | -0.072 | -0.026 |
|  | BIS |  |  | 0.233 | 0.109 | BIS |  |  | 0.221 | 0.103 |
|  | BAS-D |  |  | -0.144 | -0.071 | BAS-D |  |  | -0.167 | -0.083 |
|  | BAS-F |  |  | 0.209 | 0.089 | BAS-F |  |  | 0.235 | 0.100 |
|  | BAS-R |  |  | -0.022 | -0.009 | BAS-R |  |  | -0.009 | -0.003 |
|  |  |  |  |  |  |  |  |  |  |  |
| *Social* |  |  |  |  |  |  |  |  |  |  |
| Step 1 |  | 3.046^*^ | .037 |  |  |  | 3.046^*^ | .037 |  |  |
|  | Testing Time |  |  | 0.167 | 0.084 | Testing Time |  |  | 0.167 | 0.084 |
|  | Age |  |  | 0.045 | 0.098 | Age |  |  | 0.045 | 0.098 |
|  | Gender |  |  | -0.232 | -0.114 | Gender |  |  | -0.232 | -0.114 |
| Step 2 |  | 24.772^***^ | .308 |  |  |  | 24.772^***^ | .308 |  |  |
|  | Risk-COVID |  |  | -0.175 | -0.176^**^ | Risk-COVID |  |  | -0.175 | -0.176^**^ |
|  | Benefit-COVID |  |  | 0.570 | 0.520^***^ | Benefit-COVID |  |  | 0.570 | 0.520^***^ |
| Step 3 |  | 19.071^***^ | .081 |  |  |  | 18.959^***^ | .080 |  |  |
|  | GRiPS |  |  | 0.066 | 0.063 | GRiPS-COVID |  |  | 0.016 | 0.016 |
|  | Dohmen |  |  | 0.063 | 0.158^*^ | Dohmen |  |  | 0.078 | 0.195^**^ |
|  | Numeracy-Gen |  |  | 0.128 | 0.138^*^ | Numeracy-Gen |  |  | 0.129 | 0.140^*^ |
|  | Numeracy-Risk |  |  | 0.050 | 0.091 | Numeracy-Risk |  |  | 0.050 | 0.092 |
| Step 4 |  | 10.925^***^ | .012 |  |  |  | 10.896^***^ | .013 |  |  |
|  | BIS-AI |  |  | 0.205 | 0.098 | BIS-AI |  |  | 0.207 | 0.100 |
|  | BIS-MI |  |  | 0.038 | 0.013 | BIS-MI |  |  | 0.051 | 0.017 |
|  | BIS-NPI |  |  | -0.213 | -0.093 | BIS-NPI |  |  | -0.217 | -0.095 |
|  | BIS |  |  | -0.125 | -0.070 | BIS |  |  | -0.133 | -0.074 |
|  | BAS-D |  |  | -0.028 | -0.017 | BAS-D |  |  | -0.022 | -0.013 |
|  | BAS-F |  |  | -0.002 | -0.001 | BAS-F |  |  | 0.005 | 0.003 |
|  | BAS-R |  |  | 0.025 | 0.012 | BAS-R |  |  | 0.020 | 0.009 |
|  |  |  |  |  |  |  |  |  |  |  |
| *Health/Safety* |  |  |  |  |  |  |  |  |  |  |
| Step 1 |  | 3.974^**^ | .048 |  |  |  | 3.974^**^ | .048 |  |  |
|  | Testing Time |  |  | 0.139 | 0.058 | Testing Time |  |  | 0.139 | 0.058 |
|  | Age |  |  | 0.020 | 0.037 | Age |  |  | 0.020 | 0.037 |
|  | Gender |  |  | -0.474 | -0.195^**^ | Gender |  |  | -0.474 | -0.195^**^ |
| Step 2 |  | 19.717^***^ | .248 |  |  |  | 19.717^***^ | .248 |  |  |
|  | Risk-COVID |  |  | -0.258 | -0.220^***^ | Risk-COVID |  |  | -0.258 | -0.220^***^ |
|  | Benefit-COVID |  |  | 0.488 | 0.392^***^ | Benefit-COVID |  |  | 0.488 | 0.392^***^ |
| Step 3 |  | 17.288^***^ | .107 |  |  |  | 17.032^***^ | .103 |  |  |
|  | GRiPS |  |  | 0.425 | 0.339^***^ | GRiPS-COVID |  |  | 0.298 | 0.248^***^ |
|  | Dohmen |  |  | -0.001 | -0.002 | Dohmen |  |  | 0.061 | 0.127^*^ |
|  | Numeracy-Gen |  |  | -0.056 | -0.050 | Numeracy-Gen |  |  | -0.056 | -0.050 |
|  | Numeracy-Risk |  |  | 0.033 | 0.050 | Numeracy-Risk |  |  | 0.042 | 0.065 |
| Step 4 |  | 10.674^***^ | .030 |  |  |  | 10.579^***^ | .032 |  |  |
|  | BIS-AI |  |  | 0.322 | 0.129^*^ | BIS-AI |  |  | 0.347 | 0.139^*^ |
|  | BIS-MI |  |  | 0.132 | 0.038 | BIS-MI |  |  | 0.174 | 0.050 |
|  | BIS-NPI |  |  | 0.036 | 0.013 | BIS-NPI |  |  | -0.018 | -0.007 |
|  | BIS |  |  | 0.120 | 0.056 | BIS |  |  | 0.068 | 0.032 |
|  | BAS-D |  |  | 0.008 | 0.004 | BAS-D |  |  | 0.033 | 0.016 |
|  | BAS-F |  |  | 0.074 | 0.031 | BAS-F |  |  | 0.120 | 0.051 |
|  | BAS-R |  |  | -0.043 | -0.017 | BAS-R |  |  | -0.071 | -0.028 |
